# Supplementary material for: Perforation of the host cell plasma membrane during Toxoplasma invasion requires rhoptry exocytosis
Source: EMBO Rep. 2025 Sep 19;26(20):5027–47. doi: 10.1038/s44319-025-00564-9 (PMC12549874; doi:10.1038/s44319-025-00564-9)
Supplement: Supplementary file 1 — Appendix [file 44319_2025_564_MOESM1_ESM.pdf]

APPENDIX

Perforation of the host cell plasma membrane during *Toxoplasma* invasion requires rhoptry exocytosis

Frances Male, Yuto Kegawa, Paul S. Blank, Irene Jiménez-Munguía, Saima M. Sidik, Dylan Valleau, Sebastian Lourido, Maryse Lebrun, Joshua Zimmerberg, Gary E. Ward

Table of contents

Page 2: **Appendix Figure S1.** Gallery of the 23 individual calcium spikes combined to generate Figure 2C

Page 3: **Appendix Figure S2.** Confirmation of protein depletion in the mutant parasite lines by Western blot

Pages 4-5: **Appendix Figure S3.** The effects of rapamycin or ATc treatment on calcium transients and invasions for parental parasite lines

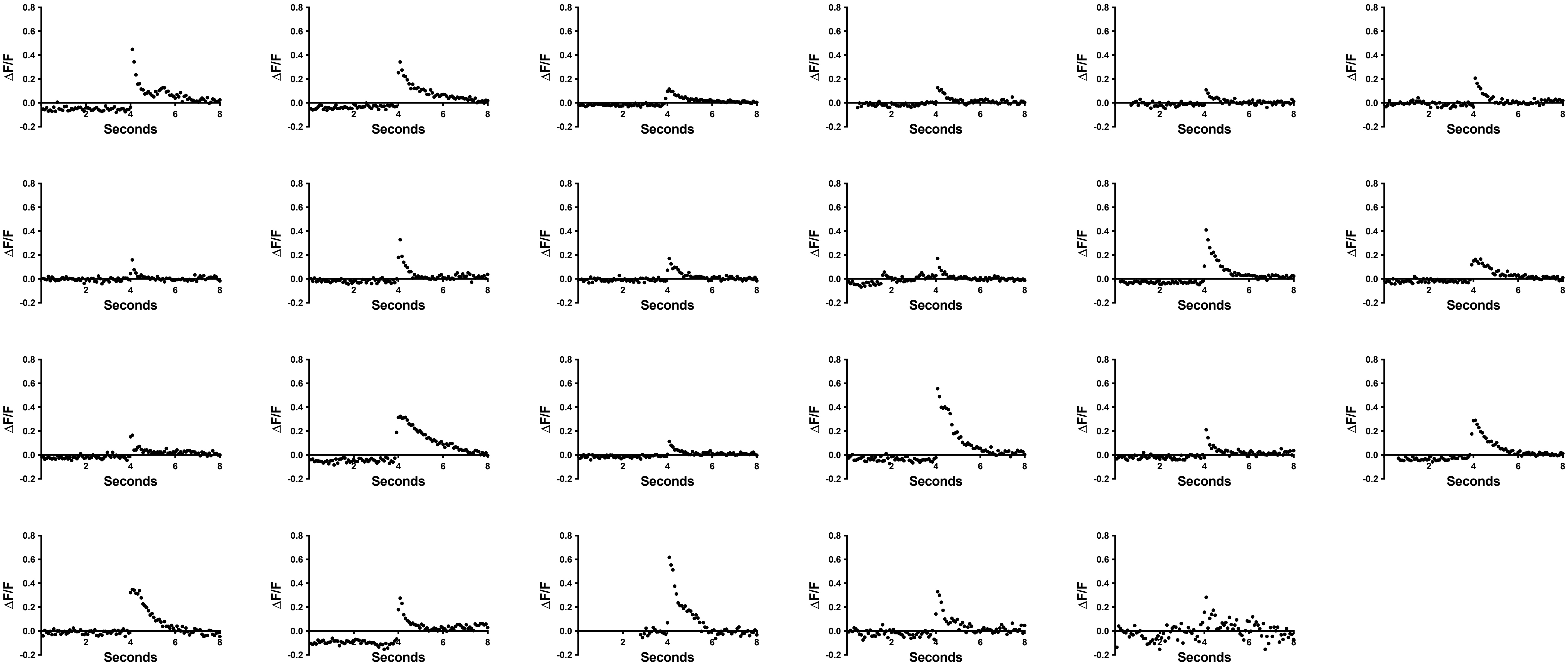

Appendix Figure S1. Gallery of the 23 individual calcium spikes combined to generate Figure 2C.

Fluo-4 fluorescence intensities (ΔF/F) in the host cell measured during 23 individual *T. gondii* invasion events are shown.

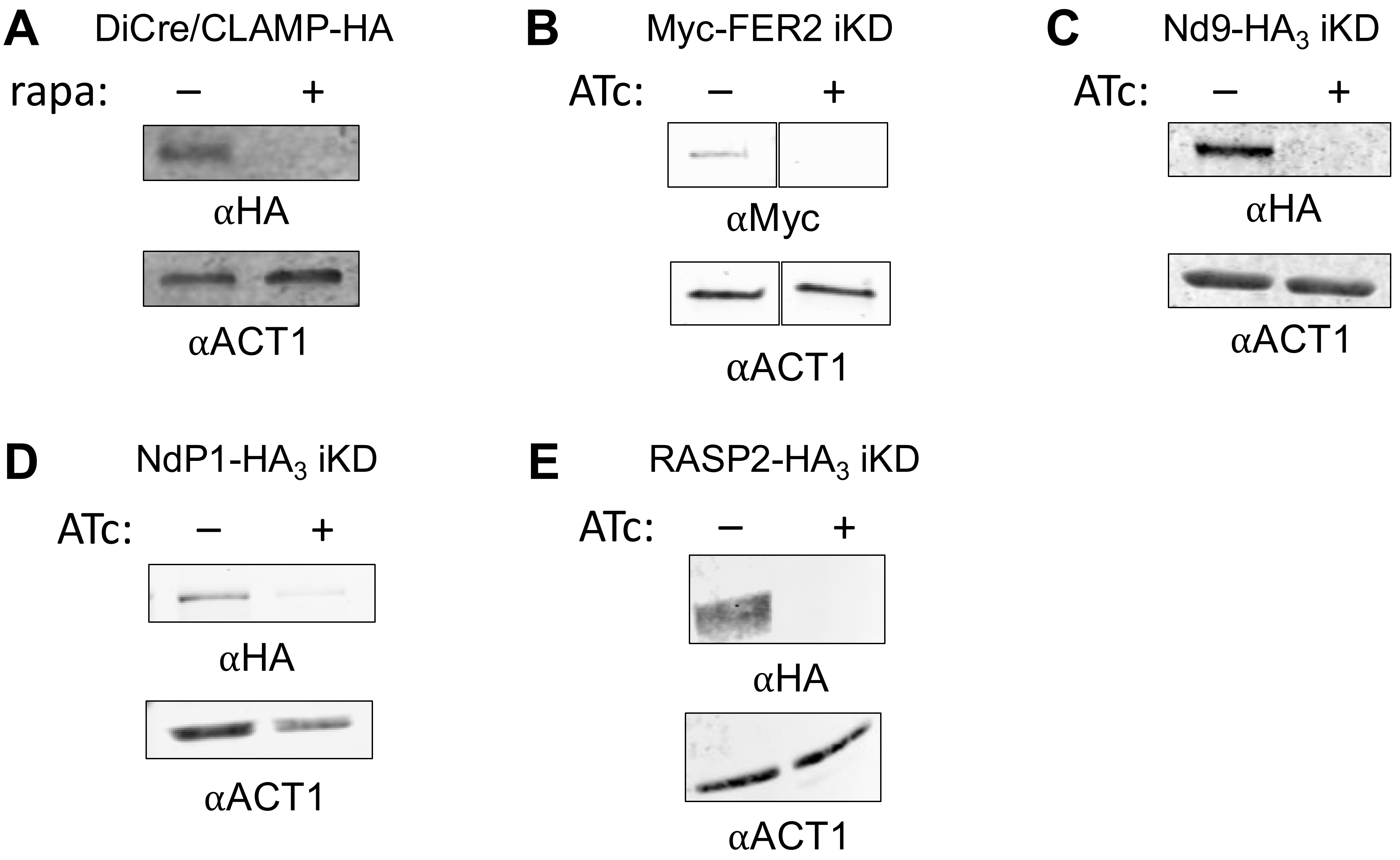

Appendix Figure S2. Confirmation of protein depletion in the mutant parasite lines by Western blot.

(A) DiCre/CLAMP-HA parasites were treated with 50 nM rapamycin (rapa; +) or an equivalent volume of DMSO (-) for 2 hours prior to 48-hour culture in drug-free medium, then total parasite lysate was analyzed by Western blot using anti-HA. Anti-actin (ACT1) was used as a loading control.

(B-E) Parasite lines were treated with 1.5 μg/mL anhydrotetracycline (ATc; +) or an equivalent volume of EtOH (-) for 96 hours (Myc-FER2 iKD (B)), 72 hours (Nd9-HA_3 iKD (C), NdP1-HA_3 iKD (D)), or 48 hours (RASP2-HA_3 iKD (E)), then total parasite lysate was analyzed by Western blot using either anti-myc or anti-HA. Anti-ACT1 was used as a loading control.

A

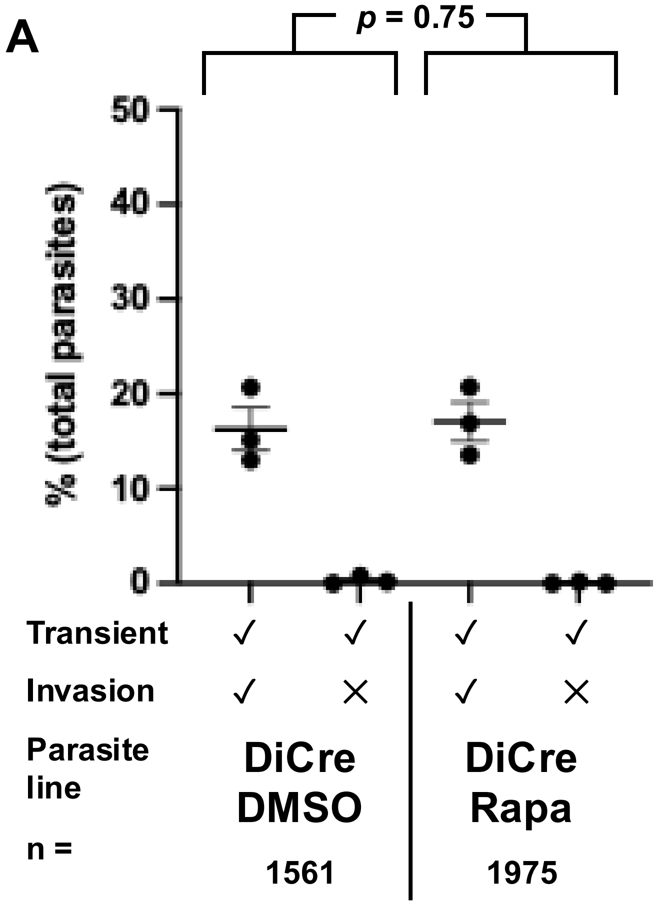

C

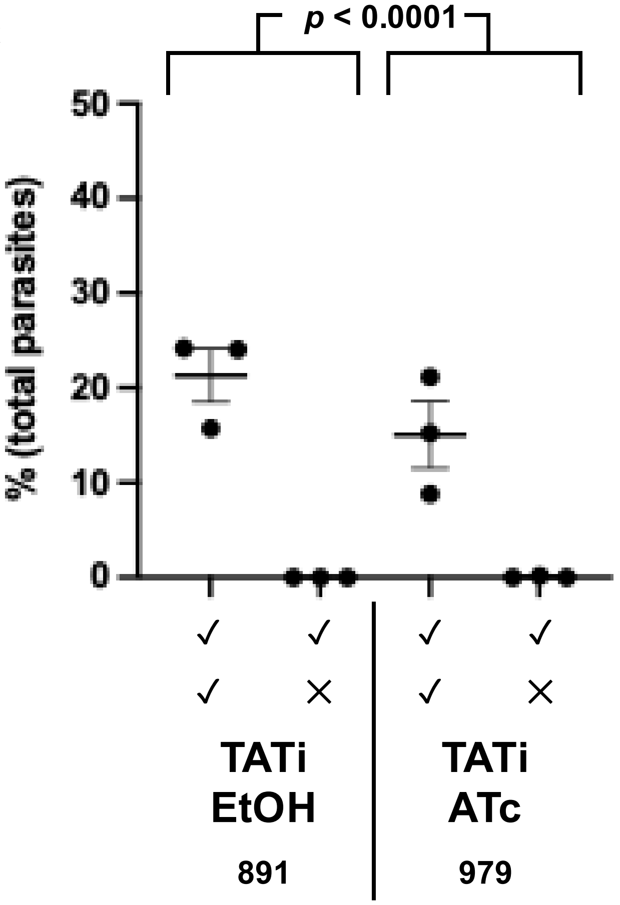

B **RH DiCre**

Treatment	+Transient +Invasion	+Transient –Invasion	–Transient +Invasion	–Transient –Invasion	Fisher's exact test
DMSO	252	3	2	1304	$p = 0.0069$
Rapamycin	330	1	17	1627	

B'

Treatment	+Transient	–Transient	Fisher's exact test	Odds ratio (95% CI)	Odds ratio P-value
DMSO	255	1306	$p = 0.75$	1.02 (0.85, 1.22)	$p = 0.838$
Rapamycin	331	1664			

D **RH TATi**

Treatment	+Transient +Invasion	+Transient –Invasion	–Transient +Invasion	–Transient –Invasion	Fisher's exact test
EtOH	178	0	38	675	$p < 0.0001$
ATc	121	1	27	830	

D'

Treatment	+Transient	–Transient	Fisher's exact test	Odds ratio (95% CI)	Odds ratio P-value
EtOH	178	713	$p < 0.0001$	0.57 (0.44, 0.73)	$p < 0.0001$
ATc	122	857			

E

Line	Odds ratio (95% CI)	Breslow-Day test (compared to TATi)	Effect size (absolute value)
TATi (Supp. Fig. 8D')	0.57 (0.44, 0.73)	--	0.311
FER2 (Supp. Fig. 3C')	0.03 (0.02, 0.06)	$p < 0.0001$	1.937
Nd9 (Supp. Fig. 3D')	0.12 (0.08, 0.17)	$p < 0.0001$	1.171
NdP1 (Supp. Fig. 3E')	0.18 (0.14, 0.24)	$p < 0.0001$	0.947
RASP2 (Supp. Fig. 3F')	0.09 (0.06, 0.14)	$p < 0.0001$	1.330

Appendix Figure S3 (previous page). The effects of rapamycin or ATc treatment on calcium transients and invasions for parental parasite lines.

(A) Quantification of invasion events and calcium transients induced by RH DiCre parental line parasites treated with DMSO (DiCre DMSO, n = 1561) compared to parasites treated with rapamycin (DiCre Rapa, n = 1975). Each data point represents one biological replicate, consisting of the average of 2-3 technical replicates; horizontal bars indicate mean ± SEM. Comparison of total calcium transients between DMSO and rapamycin groups was analyzed using Fisher's exact test, $p = 0.75$ (see panel B').

(B) Total data from DMSO-treated (top row) and rapamycin-treated (bottom row) RH DiCre parasites were categorized as in Figure EV2 A-F within a 4×2 contingency table for comparison. Fisher's exact test was used for the comparison (right-most column). Each number represents the sum of 3 biological replicates, consisting of 2-3 technical replicates each.

(B') Data from (B) were summed based on the presence or absence of detected calcium transients (+ transient, – transient) and organized into 2×2 contingency tables. Fisher's exact test and odds ratio were used for the comparison.

(C) Quantification of invasion events and calcium transients induced by RH TATi parental line parasites treated with ethanol (TATi EtOH, n = 891) compared to parasites treated with anhydrotetracycline (TATi ATc, n = 979). Each data point represents one biological replicate, consisting of the average of 2-3 technical replicates; horizontal bars indicate mean ± SEM. Comparison of total calcium transients between EtOH and ATc groups was analyzed using Fisher's exact test, $p < 0.0001$(see panel D').

(D) Total data from EtOH-treated (top row) parasites and ATc-treated (bottom row) parasites were categorized as in Figure EV2 A-F within a 4×2 contingency table for comparison. Fisher's exact test was used for the comparison (right-most column). Each number represents the sum of 3 biological replicates, consisting of 2-3 technical replicates each.

(D') Data from (D) were summed based on the presence or absence of detected calcium transients (+ transient, – transient) and organized into 2×2 contingency tables. Fisher's exact test and odds ratio were used for the comparison.

(E) Comparison of odds ratios and effect sizes between the TATi parental line (D') and each of the TATi iKD lines (FER2, Nd9, NdP1, RASP2; Figure EV2 C'-F', respectively.)
