# Supplementary figures and images for: Perforation of the host cell plasma membrane during Toxoplasma invasion requires rhoptry exocytosis
Source: EMBO Rep. 2025 Sep 19;26(20):5027–47. doi: 10.1038/s44319-025-00564-9 (PMC12549874; doi:10.1038/s44319-025-00564-9)

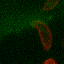

Supplement: Supplementary file 9 — Source data Fig. 2 [file 44319_2025_564_MOESM9_ESM.zip › Figure 2/2A/image2_6.32s.tiff]

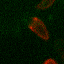

Supplement: Supplementary file 9 — Source data Fig. 2 [file 44319_2025_564_MOESM9_ESM.zip › Figure 2/2A/image4_15.04s.tiff]

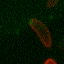

Supplement: Supplementary file 9 — Source data Fig. 2 [file 44319_2025_564_MOESM9_ESM.zip › Figure 2/2A/image3_11.04s.tiff]

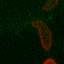

Supplement: Supplementary file 9 — Source data Fig. 2 [file 44319_2025_564_MOESM9_ESM.zip › Figure 2/2A/image1_6.08s.tiff]
