# Supplementary material for: Perforation of the host cell plasma membrane during Toxoplasma invasion requires rhoptry exocytosis
Source: EMBO Rep. 2025 Sep 19;26(20):5027–47. doi: 10.1038/s44319-025-00564-9 (PMC12549874; doi:10.1038/s44319-025-00564-9)
Supplement: Supplementary file 14 — Expanded View Figures [file 44319_2025_564_MOESM14_ESM.pdf]

Expanded View Figures

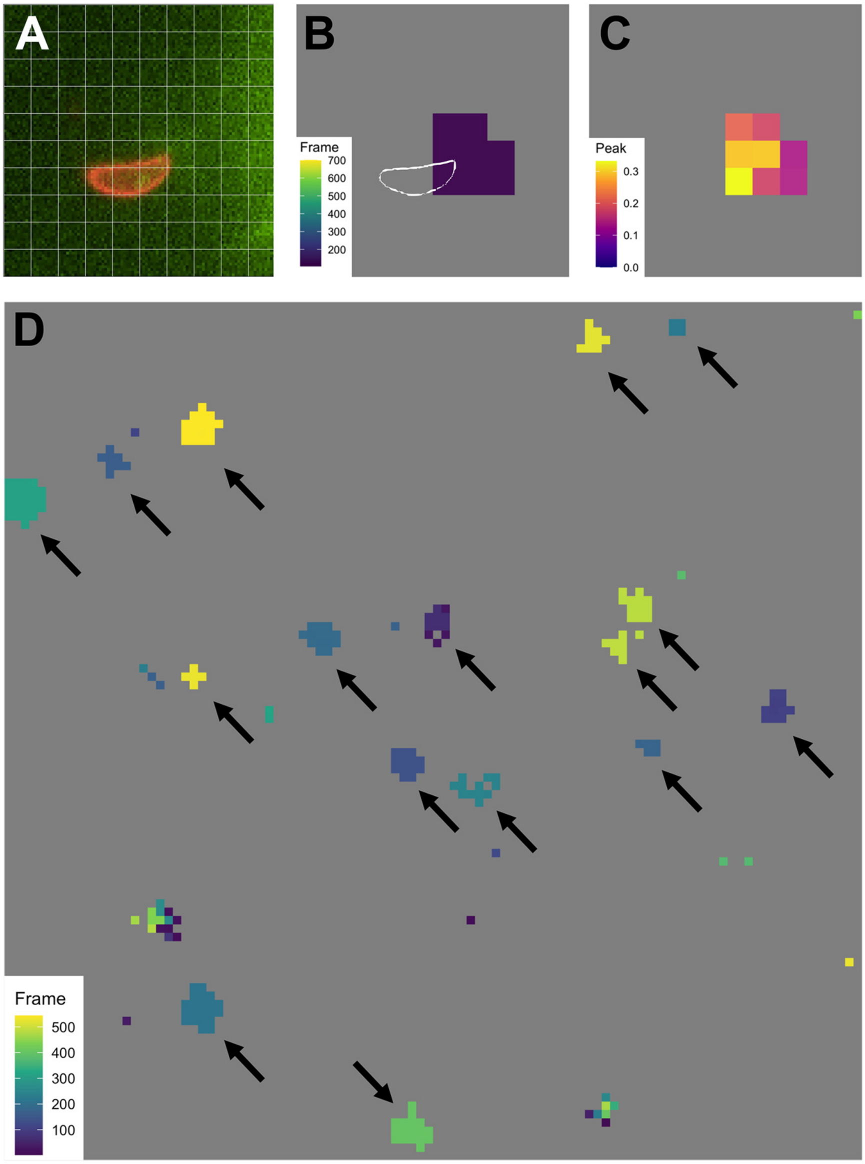

Figure EV1. Semi-automated workflow for quantifying the parasite-induced calcium transients in the host cell.

(**A**) Individual invasion event used to illustrate the quantification scheme. Mean fluorescence intensity (MFI) was captured over time from 100 10 × 10 pixel regions of interest (ROIs; white grid lines) in this 100 × 100 pixel example field of view (FOV). The full FOV for each capture consists of 1020 × 1020 pixels. (**B**) PeakCaller output plotted back to ROI location and color-coded by frame (inset) at which the transient event reached maximal intensity; location of parasite outlined in white. This transient reached its peak at frame 106 of 700 frames total. (**C**) PeakCaller output plotted back to ROI location and color-coded by peak intensity (inset) achieved during the transient event. (**D**) Sixteen calcium transients (black arrows) were detected in this representative full FOV (221 × 221 µm) over the course of 48 s of recording (600 frames). Full captures consist of 1200 frames; fewer frames are shown here for clarity. MFI was captured over time from 10,404 10 × 10 pixel ROIs across 1020 × 1020 pixel FOV. PeakCaller output was plotted back to ROI location and color-coded by the frame (inset) when each transient reached peak intensity.

 © The Author(s)

A WT (RH)

Treatment	+Transient +Invasion	+Transient –Invasion	–Transient +Invasion	–Transient –Invasion
N/A	206	3	9	836

B CLAMP

Treatment	+Transient +Invasion	+Transient –Invasion	–Transient +Invasion	–Transient –Invasion	Fisher's exact test
DMSO	521	9	57	1934	$p < 0.0001$
Rapamycin	113	1	11	2412	

B'

+Transient	–Transient	Fisher's exact test
530	1991	$p < 0.0001$
114	2423	

C FER2

Treatment	+Transient +Invasion	+Transient –Invasion	–Transient +Invasion	–Transient –Invasion	Fisher's exact test
EtOH	436	3	21	1165	$p < 0.0001$
ATc	11	4	1	1852	

C'

+Transient	–Transient	Fisher's exact test
439	1186	$p < 0.0001$
15	1853	

D Nd9

Treatment	+Transient +Invasion	+Transient –Invasion	–Transient +Invasion	–Transient –Invasion	Fisher's exact test
EtOH	261	1	56	711	$p < 0.0001$
ATc	10	16	7	654	

D'

+Transient	–Transient	Fisher's exact test
262	767	$p < 0.0001$
26	661	

E NdP1

Treatment	+Transient +Invasion	+Transient –Invasion	–Transient +Invasion	–Transient –Invasion	Fisher's exact test
EtOH	260	1	14	708	$p < 0.0001$
ATc	42	29	6	1091	

E'

+Transient	–Transient	Fisher's exact test
261	722	$p < 0.0001$
71	1097	

F RASP2

Treatment	+Transient +Invasion	+Transient –Invasion	–Transient +Invasion	–Transient –Invasion	Fisher's exact test
EtOH	384	2	23	1336	$p < 0.0001$
ATc	18	6	0	902	

F'

+Transient	–Transient	Fisher's exact test
386	1359	$p < 0.0001$
24	902	

Figure EV2. Total invasion and transient counts for each parasite line analyzed and contingency table analysis for the inducible knockdown parasites.

(**A**) Total WT parasite counts were separated into four categories: +transient/+invasion; +transient/−invasion; −transient/+invasion; and −transient/−invasion. Each number represents the sum of three biological replicates, consisting of two to three technical replicates each. (**B**−**F**) Data from all mutant parasite lines categorized as in (**A**) within 4 × 2 contingency tables to compare control (top row) and protein-depleted (bottom row) parasites. Each number represents the sum of three biological replicates, consisting of two to three technical replicates each. Fisher's exact test was used for the comparison (right-most column). (**B′**−**F′**) Data from each mutant line (**B**−**F**) were summed based on the presence or absence of detected calcium transients (+transient, −transient) and organized into 2 × 2 contingency tables. Fisher's exact test was used for the comparison (right-most column).

A

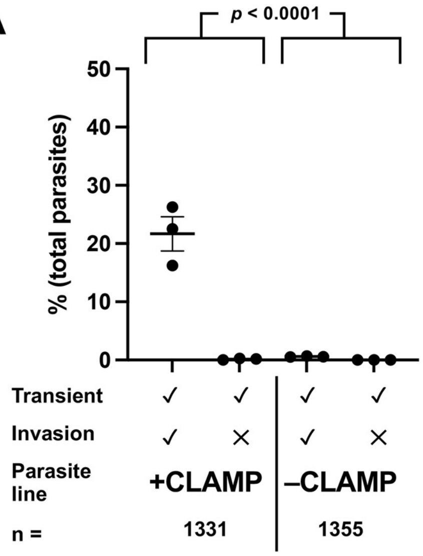

B CLAMP

Treatment	+Transient +Invasion	+Transient –Invasion	–Transient +Invasion	–Transient –Invasion	Fisher's exact test
DMSO	284	2	1	1044	$p < 0.0001$
Rapamycin	8	0	0	1347	

B'

Treatment	+Transient	–Transient	Fisher's exact test
DMSO	286	1045	$p < 0.0001$
Rapamycin	8	1347	

C D E F

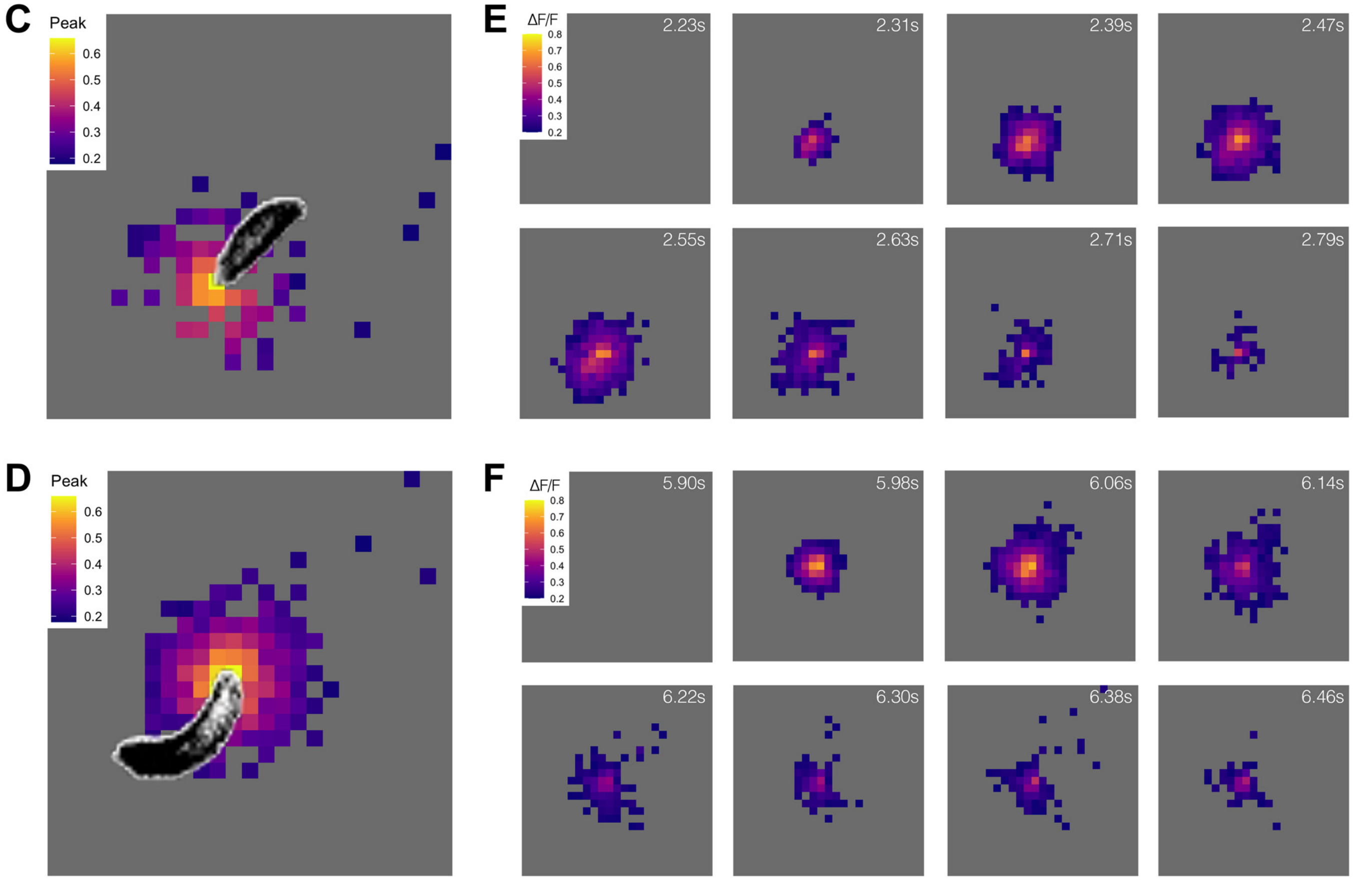

 © The Author(s)

Figure EV3. Improved detection of calcium transients.

(**A**) Quantification of invasion events and calcium transients induced by control parasites (+CLAMP, $n = 1331$) compared to parasites depleted of CLAMP (−CLAMP, $n = 1355$) using a fluorescence detection method with increased sensitivity (Cal-520, probenecid treatment, 8 mM extracellular $CaCl_2$; see text for details). Each data point represents one biological replicate, consisting of the average of two to three technical replicates; horizontal bars indicate mean ± SEM. Comparison of total calcium transients between +CLAMP and −CLAMP groups was analyzed using Fisher's exact test, $p < 0.0001$ (Fig. EV3B'). (**B**) Total data from control-treated (top) parasites and CLAMP-depleted (bottom) parasites were categorized as in Fig. EV2A–F within a 4 × 2 contingency table for comparison. Fisher's exact test was used for the comparison (right-most column). Each number represents the sum of three biological replicates, consisting of three technical replicates each. (**B'**) Data from (**B**) were summed based on the presence or absence of detected calcium transients (+transient, –transient) and organized into 2 × 2 contingency tables. Fisher's exact test was used for the comparison (right-most column). (**C–F**) Panels (**C**, **E**) display the calcium transient shown in Movie EV4, after binning the data into 4 × 4 pixel ROIs (0.87 × 0.87 μm). Panel (**C**) shows the PeakCaller output based on mean fluorescence intensity within each ROI, color coded by peak intensity (see Fig. EV1 for PeakCaller methods). The overlay shows that location of the parasite at the initiation of invasion. Panel E shows the same transient, but in this case the change in fluorescence intensity within each ROI was divided by the median intensity within the ROI over the entire time series (ΔF/F). Time points represent consecutive frames and time is shown in seconds (s). Panels (**D**, **F**) display analysis of a second calcium transient (Movie EV5), analyzed identically to the data in panels (**C**, **E**).

© The Author(s)

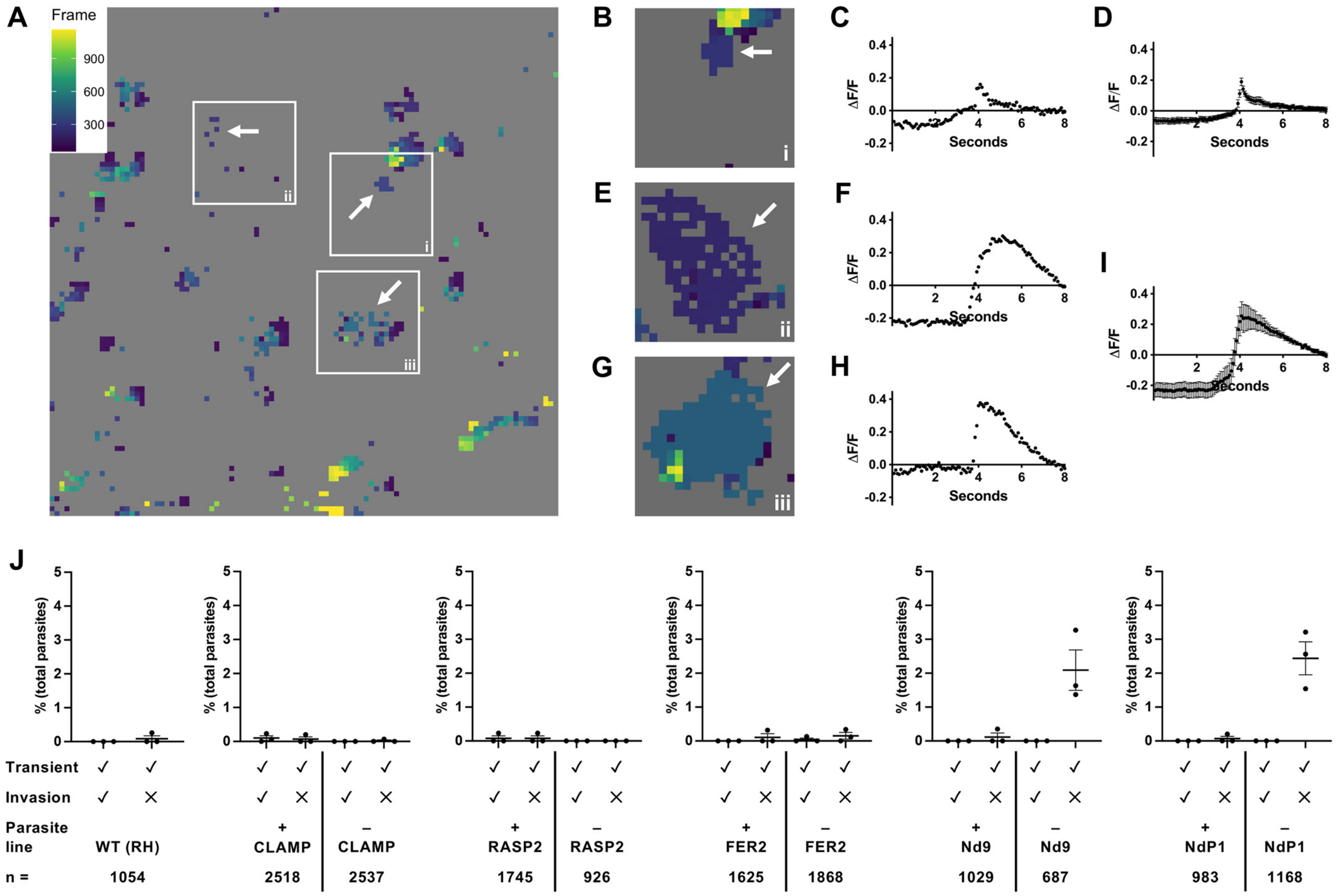

Figure EV4. Aberrant calcium transients detected in all parasite lines tested are typically associated with non-invading parasites.

(**A**) Calcium transients generated by NdP1-depleted parasites. The PeakCaller output from the full 1020 × 1020 pixel FOV was plotted back to ROI location and color-coded by frame (inset) at which the transients reached their maximal intensity. Box i highlights a calcium transient associated with an invading parasite and boxes ii and iii highlight two aberrant calcium transients associated with parasites that subsequently failed to invade. (**B**, **E**, **G**) Magnified views of the fluorescence signals detected in boxes i–iii from (**A**), after reanalyzing the data with parameters optimized for capture of aberrant transient events. (**C**, **F**, **H**) Quantification of Fluo-4 fluorescence levels (ΔF/F) in the host cell during the calcium transient events shown in (**B**, **E**, **G**) respectively. (**D**, **I**) Consensus plot of calcium transients generated by NdP1-depleted parasites that subsequently invaded the host cell ((**D**), $n = 15$) and NdP1-depleted parasites that did not subsequently invade ((**I**), $n = 9$). The fluorescence intensities in the 100 frames surrounding the peak of each calcium transient were averaged across all transients, the peaks of which were aligned to frame 51. The plot shows the mean ± SEM at each time point. (**J**) Quantification of the frequency of aberrant spike generation by each of the parasite lines analyzed in this study. Each graph shows the number of aberrant spikes generated, as a percentage of the total number of parasites counted, and whether the aberrant spikes were associated with invading or non-invading parasites. Each data point represents one biological replicate, consisting of the average of two to three technical replicates. Horizontal bars indicate mean ± SEM.

 © The Author(s)

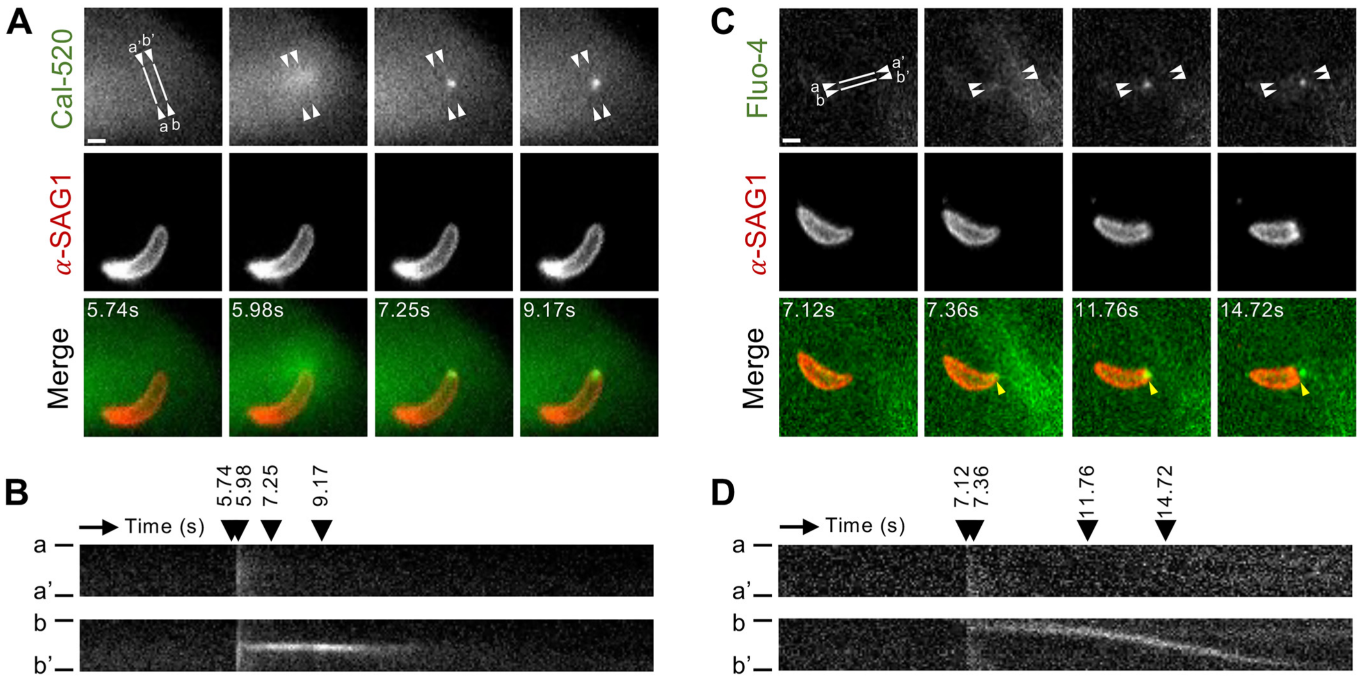

Figure EV5. Development of a dot of fluorescence at the apical end of parasites invading calcium indicator-loaded host cells.

Cal-520 (**A**) or Fluo-4 (**C**) fluorescence (top row) during invasion of calcium indicator-loaded host cells by anti-SAG1-labeled parasites (middle row). Note the appearance of a dot of fluorescence at the parasite apex. Scale bar = 2 μm; time is shown in seconds (s). Yellow arrowheads in (**C**) indicate the position of the moving junction. The full videos from which these images were extracted are presented as Movies EV5 and EV3, respectively. Panels (**B**, **D**) show the kymographs of calcium indicator fluorescence intensity, captured over time along the lines (a to a′ and b to b′) indicated in Panels (**A**, **C**). For both, a to a′ is a line through a control region of the cell, while b to b′ captures the appearance/movement of the dot. In kymograph (**B**) the calcium transient occurs at 5.98 s and precedes the formation and intensification of the dot of fluorescence that is evident along line b to b′. In kymograph (**D**), the calcium transient is evident at 7.36 s, and the subsequent movement of the fluorescent dot at the apical tip of the parasite past the moving junction (yellow arrowhead) and into the host cell is evident along line b to b′.

© The Author(s)
